# Protective Effect of *Salvia miltiorrhiza* Extract Against Renal Ischemia-Reperfusion-Induced Injury in Rats

**DOI:** 10.3390/molecules17021191

**Published:** 2012-01-30

**Authors:** Gang Chen, Yunrui Fu, Xiaohou Wu

**Affiliations:** Department of Urology, The First Affiliated Hospital, Chongqing Medical University, Chongqing 400016, China; Email: gangc4651@sina.com (G.C.); yrfu81@sina.com (Y.F.)

**Keywords:** *Salvia miltiorrhiza* ethanol extracts, ischemia reperfusion, Scr, BUN, antioxidant

## Abstract

The present study investigates the effect of pre-treatment with *Salvia miltiorrhiza* ethanol extracts (SMEE) on renal function markers, immunity and antioxidant activities in renal ischemia and reperfusion (IR) rats. Wistar rat kidneys were subjected to 60 min of global ischemia at 37 °C followed by 30 min of reperfusion, and were randomly assigned into the sham, IR model and three SMEE-treated groups (n = 8 per group). Results showed that high serum creatinin (Scr), blood urea nitrogen (BUN), interleukin-6 (IL-6), interleukin-8 (IL-8), tumor necrosis factor-alpha (TNF-α) and malondialhehyde (MDA) levels, and low antioxidant enzyme activities were observed in IR rats compared to the sham rats. Pre-treatment of Salvia miltiorrhiza ethanol extracts for 20 days prior to IR operation improved renal function, reduced IR induced renal inflammatory and oxidative injury. It is concluded that *Salvia miltiorrhiza* ethanol extracts could be beneficial in the treatment of renal ischemic injury.

## 1. Introduction

Renal tissue ischemia-reperfusion (I/R) injury can occur in some important clinical situations such as severe hypotension and subsequent resuscitation, kidney transplantation and aortovascular surgeries, that can all lead to acute renal failure (ARF) [1].

A main event in the induction of kidney injury during I/R is the generation of reactive oxygen species (ROS) [2,3]. ROS, which are produced during renal reperfusion, have diverse cytotoxic effects, including DNA damage, protein oxidation and nitrosylation, lipid peroxidation, and induction of apoptosis [4]. I/R injury to the kidney also causes endothelial dysfunction and local inflammatory responses [5]. Evidence of oxygen radical-mediated injury in the kidney includes demonstration of renal injury being accentuated by oxidants and the observation that deficiency of antioxidants exacerbates renal injury and that free radical-mediated lipid peroxidation occurs as a manifestation of ischemia-reperfusion injury also implicate oxidants in the pathophysiology of acute renal failure [6,7]. The protective effect of supplemention with antioxidant enzymes [8,9,10,11,12] and transgenic animals [13] and cells [14] supplemented with antioxidant gene/enzyme against ischemia-reperfusion induced oxidative stress provide unequivocal evidence that antioxidant enzymes impact on the degree of tissue damage.

A well known medicinal plant Radix Salvia miltiorrhiza (‘Dansham’ in Korean and ‘Danshen’ in Chinese), the root of *Salvia miltiorrhiza* Bunge (Labiatae), has been used in Chinese folk medicine for the treatment of coronary heart diseases, renal diseases, myocardial infarction, and hypertension [15,16]. In a survey of the relevant literature, strong protective action, against oxidative damage to liver microsomes, hepatocytes and erythrocytes, had been demonstrated for seven phenolic compounds isolated from *S. miltiorrhiza* as active components [17]. The purpose of this study was to investigate the effects of SMEE on Scr, BUN, IL-6, IL-8, TNF-α, SOD, GSH-Px and catalase activities and GSH and MDA levels in ischemia and reperfusion rat renal tissue.

## 2. Results

The blood urea nitrogen and serum creatinine levels in the I/R group were found to be significantly higher than those in the sham rats (*p* < 0.01; Table 1). When *Salvia miltiorrhiza* ethanol extract (50, 100 and 150 mg·kg^−1^ bw) and tanshinone (25 mg/kg b.w.) were administered before ischemia and the subsequent reperfusion period, although these levels were still significantly higher than the sham control, the elevation in BUN and serum creatinine levels were significantly depressed (*p* < 0.01). In addition, serum Scr, and BUN levels in experimental rats fed with *Salvia miltiorrhiza* ethanol extract (150 mg·kg^−1^ bw) were significantly (*p* < 0.05) lower than ones in sham rats.

In the I/R group renal IL-6 contents (3.83 ± 0.12 ng/mL) were found to be significantly higher than that of sham control group (1.51 ± 0.18 ng/mL). *Salvia miltiorrhiza* ethanol extract (50, 100 and 150 mg·kg^−1^ bw) and tanshinone (25 mg/kg b.w.) pretreatment for 20 days significantly reduced this parameter in the I/R + SMEE groups (2.09 ± 0.18, 1.79 ± 0.14, 1.52 ± 0.17 and 1.82 ng/mL) which is close to sham control values (Table 1). In addition, renal IL-6 levels in experimental rats fed with *Salvia miltiorrhiza* ethanol extract (150 mg·kg^−1^ bw) were slightly (*p* > 0.05) lower than ones in sham rats.

**Table 1:** Effect of *Salvia miltiorrhiza* ethanol extract pretreatment on serum Scr, BUN, renal IL-6, IL-8 and TNF-α levels in experimental rats.

Group	Scr (mmol/L)	BUN (mmol/L)	IL-6 (ng/mL)	IL-8 (ng/mL)	TNF-α (ng/mL)
sham	68.18 ± 3.48	6.29 ± 0.42	1.51 ± 0.18	1.42 ± 0.25	0.514 ± 0.038
SMEE (150 mg/kg b.w.)	60.27 ± 2.74 ^a^	5.45 ± 0.32 ^a^	1.49 ± 0.12	1.31 ± 0.19	0.431 ± 0.024 ^a^
I/R model	174.92 ± 10.73 ^b^	13.74 ± 1.04 ^b^	3.83 ± 0.12 ^b^	5.97 ± 0.52 ^b^	0.922 ± 0.081 ^b^
I/R+SMEE (50 mg/kg b.w.)	129.11 ± 8.04 ^d^	10.13 ± 0.71 ^d^	2.09 ± 0.18 ^d^	3.68 ± 0.44 ^d^	0.739 ± 0.063 ^d^
I/R+SMEE (100 mg/kg b.w.)	97.02 ± 4.77 ^d^	7.72 ± 0.66 ^d^	1.79 ± 0.14 ^d^	2.44 ± 0.27 ^d^	0.581 ± 0.066 ^d^
I/R+SMEE (150 mg/kg b.w.)	88.52 ± 7.29 ^d^	6.89 ± 0.72 ^d^	1.52 ± 0.17 ^d^	1.53 ± 0.25 ^d^	0.531 ± 0.071 ^d^
I/R+tanshinone (25 mg/kg b.w.)	99.21 ± 5.39 ^d^	7.25 ± 0.49 ^d^	1.82 ± 0.13 ^d^	2.66 ± 0.22 ^d^	0.605 ± 0.053 ^d^

Rats (n = 8 per group) in IR+SMEE groups were administered *Salvia miltiorrhiza* ethanol extract orally at daily doses of 50 mg/kg, 100 mg/kg and 150 mg/kg, respectively, for up to 20 days prior to IR process; Rats (n = 8 per group) in sham and IR groups were administered orally with a equal volume of vehicle (saline) for up to 20 days prior to IR process; ^a^ *P *< 0.05, ^b^ *P *< 0.01, compared with sham group; ^d^ *P *< 0.01, compared with IR group.

As shown in Table 1, the renal IL-8 level was augmented significantly in rats subjected to ischemia and reperfusion process. However, oral administration of *Salvia miltiorrhiza* ethanol extract (50, 100 and 150 mg·kg^−1^ bw) and tanshinone (25 mg/kg b.w.) for 20 days reduced the IL-8 level significantly in a dose-dependent manner. Precisely, it decreased from 5.97 to 3.68 ng/mL (38% decrease) and to 2.44 ng/mL (59% decrease) and to 1.53 ng/mL (74% decrease) and to 2.66 ng/mL (55% decrease) after doses of 50, 100, 150 and 25 mg/kg body weight/day, respectively. In addition, renal IL-8 levels in experimental rats fed with *Salvia miltiorrhiza* ethanol extract (150 mg·kg^−1^ bw) were slightly (*p* > 0.05) lower than ones in sham rats.

As shown in Table 1, the level of renal TNF-α increased significantly in the rats subjected to the ischemia and reperfusion process, in comparison with sham rats. The level of renal TNF-α were, however, lower in the rats given *Salvia miltiorrhiza* ethanol extract (50, 100 and 150 mg·kg^−1^ bw) and tanshinone (25 mg/kg b.w.) for 20 days prior to the ischemia-reperfusion process. In addition, renal TNF-α levels in experimental rats fed with *Salvia miltiorrhiza* ethanol extract (150 mg·kg^−1^ bw) were significantly (*p* < 0.01) lower than ones in sham rats.

Ischemia and reperfusion caused significant decreases in tissue GSH levels (1.02 ± 0.09 μmol/g, *p* < 0.01) when compared with the sham control group (2.55 ± 0.14 μmol/g). In the I/R+SMEE and tanshinone (25 mg/kg b.w.) groups, GSH levels were found to be dose-dependently significantly increased (*p* < 0.01) compared to IR model group (Table 2). In addition, renal GSH levels in experimental rats fed with *Salvia miltiorrhiza* ethanol extract (150 mg·kg^−1^ bw) were significantly (*p* < 0.01) higher than ones in sham rats.

The level of renal MDA was significantly increased in IR model rats compared with sham rats as shown in Table 2. In SMEE-pretreated and tanshinone (25 mg/kg b.w.)-pretreated rats, level of renal MDA was dose-dependently significantly decreased (*p* < 0.01) in comparison with IR model rats (Table 2). In addition, renal MDA levels in experimental rats fed with *Salvia miltiorrhiza* ethanol extract (150 mg·kg^−1^ bw) were significantly (*p* < 0.01) lower than ones in sham rats.

**Table 2:** Effect of *Salvia miltiorrhiza* ethanol extract pretreatment on renal MDA level in experimental rats.

Group	GSH (μmol/g protein)	MDA (nmol/mg protein)
sham	2.55 ± 0.14	4.27 ± 0.31
SMEE (150 mg/kg b.w.)	3.84 ± 0.19 ^b^	3.05 ± 0.26 ^b^
I/R model	1.02 ± 0.09 ^b^	8.06 ± 0.48 ^b^
I/R+SMEE (50 mg/kg b.w.)	1.52 ± 0.18 ^d^	6.83 ± 0.24 ^c^
I/R+SMEE (100 mg/kg b.w.)	1.99 ± 0.13 ^d^	5.74 ± 0.28 ^d^
I/R+SMEE (150 mg/kg b.w.)	2.37 ± 0.17 ^d^	4.52 ± 0.31 ^d^
I/R+tanshinone (25 mg/kg b.w.)	1.86 ± 0.13 ^d^	6.08 ± 0.37 ^d^

Rats (n = 8 per group) in IR+SMEE groups were administered *Salvia miltiorrhiza* ethanol extract orally at daily doses of 50 mg/kg, 100 mg/kg and 150 mg/kg, respectively, for up to 20 days prior to IR process; Rats (n = 8 per group) in sham and IR groups were administered orally with a equal volume of vehicle (saline) for up to 20 days prior to IR process; ^b^ *P *< 0.01, compared with sham group; ^c^ *P *< 0.05, ^d^ *P *< 0.01, compared with IR group.

As expected, rats in the I/R model group showed statistically significant decreases in renal antioxidant enzyme activities (SOD, CAT, GSH-Px) when compared to the sham group. There was a marked increase in the renal antioxidant enzyme activities (SOD, CAT, GSH-Px) in I/R+SMEE groups, pre-treated with 50, 100 and 150 mg·kg^−1^ bw *Salvia miltiorrhiza* ethanol extract and tanshinone (25 mg/kg b.w.), as compared to IR model group (Table 3). SMEE includes in its composition molecules able to scavenge oxidative species impeding a decrease in the basal concentration of antioxidant defenses. In addition, renal antioxidant enzyme activities (SOD, CAT, GSH-Px) in experimental rats fed with *Salvia miltiorrhiza* ethanol extract (150 mg·kg^−1^ bw) were significantly (*p* < 0.01) higher than ones in sham rats.

**Table 3:** Effect of *Salvia miltiorrhiza* ethanol extract pretreatment on renal SOD, CAT and GSH-Px activities in experimental rats.

Group	SOD	CAT	GSH-Px
sham	167.2 ± 11.7	43.69 ± 3.19	39.77 ± 1.88
SMEE (150 mg/kg b.w.)	208.4 ± 18.5 ^b^	58.21 ± 2.97 ^b^	51.63 ± 2.89 ^b^
I/R model	85.1 ± 3.9 ^b^	26.13 ± 2.05 ^b^	20.14 ± 1.57 ^b^
I/R+SMEE (50 mg/kg b.w.)	108.5 ± 8.4 ^c^	33.08 ± 1.77 ^c^	28.41 ± 1.93 ^c^
I/R+SMEE (100 mg/kg b.w.)	143.7 ± 13.5 ^d^	40.61 ± 3.24 ^d^	35.09 ± 2.51 ^d^
I/R+SMEE (150 mg/kg b.w.)	170.6 ± 15.7 ^d^	49.65 ± 2.91 ^d^	40.11 ± 2.67 ^d^
I/R+tanshinone (25 mg/kg b.w.)	150.3 ± 11.3 ^d^	39.06 ± 1.86 ^d^	33.79 ± 1.85 ^d^

Rats (n = 8 per group) in IR+SMEE groups were administered *Salvia miltiorrhiza* ethanol extract orally at daily doses of 50 mg/kg, 100 mg/kg and 150 mg/kg, respectively, for up to 20 days prior to IR process; Rats (n = 8 per group) in sham and IR groups were administered orally with a equal volume of vehicle (saline) for up to 20 days prior to IR process; ^b^ *P *< 0.01, compared with sham group; ^c^ *P *< 0.05, ^d^ *P *< 0.01, compared with IR group.

**Figure 1:**
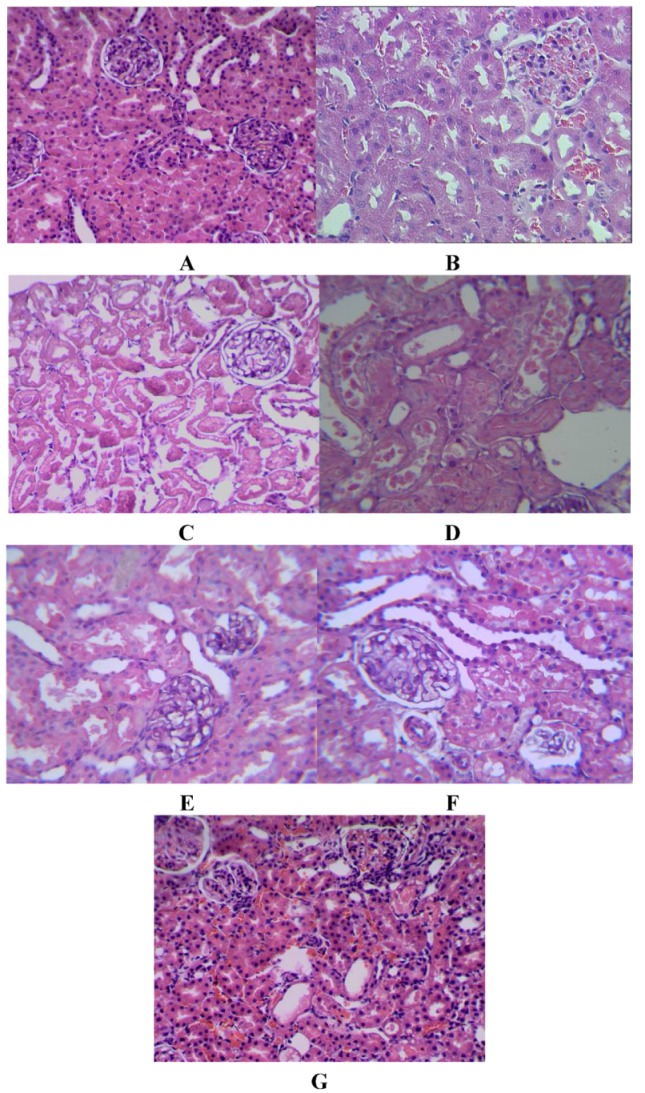
The microscopic evaluation of kidney tissue.

Light microscopic evaluation of the kidneys in the control group showed regular morphology of renal parenchymal with well-defined glomeruli and tubuli (Figure 1A,B). In the I/R group, there are significant renal interstitial hyperemia and hemorrhage, associated with inflammatory cell infiltration; intertubular interstitium showed severe detachments and edema; nuclear chromatin margination was shown in epithelial cells, as well as cytoplasmic vacuolar degeneration and necrosis; there was cellular debris in the proximal tubuli. In most areas the glomeruli had lost their morphology (Figure 1C). In the I/R+SMEE and I/R+tanshinone groups, intertubular interstitium revealed a better morphology of the glomeruli when compared with I/R group, and the severe hemorragia was no longer present (Figure 1D–G).

## 3. Discussion

Renal I/R injury is encountered in many clinical situations: transplantation, partial nephrectomy, sepsis, hydronephrosis, or elective urologic operations. It is the second greatest cause of organ failure after immunological graft rejection. Renal ischemia as a consequence of arterial occlusion, shock, and organ transplantation is a common cause of renal cell death, renal failure, delayed graft function, and renal graft rejection [18]. I/R injuries induce inflammatory responses and production of reactive oxygen species, which affect the organs remote to the sites of I/R. Previous reports indicated that renal I/R can lead to distant organ dysfunction in addition to inflammation in the renal tissues resulting in local tissue injury [19].

The acute renal failure indicated by increased Scr and BUN occurred before the development of tubular necrosis. These parameters are markers of glomerular filtration rate [20]. In this study, Scr and BUN levels in I/R rats were significantly higher than those in sham rats. This indicated that renal dysfunction occurred after I/R operation. Our results showed that pre-treatment of *Salvia miltiorrhiza* ethanol extract reduced the rise of BUN and Scr induced by ischemia reperfusion operation. This showed that pre-treatment of *Salvia miltiorrhiza* ethanol extract was helpful in preventing IR-induced renal dysfunction.

Some studies showed that inflammatory cytokine played a important role in renal ischemic injury [21,22,23]. IL-6 is a major regulator of inflammation. Furthermore, IL-6 production may be a common feature of ischemic injury of any organ. IL-6 not only is found after ischemia of the brain [24], gut [25], and heart [26], but the amount of IL-6 also correlates with the amount of ischemic injury [27]. Interleukin-8 (IL-8) is a chemokine produced by macrophages and other cell types such as epithelial cells. It is also synthesized by endothelial cells, which store IL-8 in their storage vesicles, the Weibel-Palade bodies [28,29]. TNF-α reduces glomerular perfusion by inducing the synthesis of vasoconstrictive and vasodilatory mediators [30]. Higher levels of renal IL-8, IL-6 and TNF-α were found in the IR group compared with the sham group. This indicated that IR rats suffered from renal Inflammatory injury. Lower levels of renal IL-8, IL-6 and TNF-α were found in the IR+SMEE group compared with the IR group. This indicated that pre-treatment with *Salvia miltiorrhiza* ethanol extract could raduce I/R-induced renal inflammatory injury.

Free radicals have been shown to play a major role in I/R [31]. ROS collectively are instrumental in impairing overall renal function [32,33,34] and in inducing apoptosis in renal cells [35]. Antioxidant therapy has been well documented to help in the improvement of organ functions [36] and to prevent apoptosis [37,38]. The protection provided by free radical scavengers against ROS produced during I/R supports the hypothesis that free radical species are involved in the cellular pathogenesis of I/R [39].

We assessed the potential of *Salvia miltiorrhiza* ethanol extract by studying its effect on lipid peroxidation, which was measured in terms of MDA, a stable metabolite of the free radical-mediated lipid peroxidation cascade. MDA levels increased with renal ischemia reperfusion process. *Salvia miltiorrhiza* ethanol extract reversed the increase of MDA levels to a considerable extent, thereby confirming its antioxidant role in I/R, indicating that *Salvia miltiorrhiza* ethanol extract prevented lipid peroxidation and protein oxidation in the renal I/R process. Scaduto *et al.* studied the effect of *Salvia miltiorrhiza* ethanol extract on yet another cellular bio-antioxidant, GSH, which is known to be depleted following an ischemic insult [40]. Similarly, in our studies, GSH was decreased with renal ischemia reperfusion process. SMEE-pretreated rats exhibited higher GSH contents than their respective controls, indicating that *Salvia miltiorrhiza* ethanol extract helped in replenishing the GSH pool. The fact that *Salvia miltiorrhiza* ethanol extract causes a significant increase in CAT, GSH-Px and SOD activities in comparison with I/R group, suggesting that it might have an antioxidant effect through the increase in SOD, GSH-Px and CAT enzyme activities.

## 4. Experimental

### 4.1. Plant Material

The roots of *Salvia miltiorrhiza* were collected from a local herb shop, Chongqing City, China in June 2010.

### 4.2. Extraction Method

The roots (200 g) of *Salvia miltiorrhiza* were powdered and extracted with ethanol (1,000 mL) under reflux for 3 h for extraction (twice). The combined extracts were evaporated under reduced pressure to give concentrated *Salvia miltiorrhiza* ethanol extract. The extract was stored in a refrigerator and maintained at 4 °C until use.

### 4.3. Animals

Forty Wistar rats (220–250 g) were selected for the study and randomly divided into five equal groups: sham group, IR group and three SMEE-treatment groups. Animal care and experiments conformed with the Guide for the Care and Use of Laboratory Animals of China and approval of the ethics committee of Chongqing Medicinal University was obtained before the commencement of the study.

The animals were housed under standard environmental conditions (23 ± 1 °C, with 55 ± 5% humidity and a 12 h light/12 h dark cycle) and maintained with free access to water and a standard laboratory diet *ad libitum*. Animals were orally administered *Salvia miltiorrhiza* ethanol extract (50, 100 or 150 mg/kg b.w.) or tanshinone (25 mg/kg b.w.) or a control vehicle (saline) daily for 20 days. Animals were anesthetized by pentothal (50 mg/kg, i.p.; Abbott Laboratories). Autoclave-sterilized surgical instruments were used for the procedure. A laparotomy was performed with a vertical midline incision, and the renal artery was exposed by blunt dissection. A hemostatic micro clamp was applied on the renal artery of the kidney for 60 min to create complete renal ischemia. The clamp was removed later to allow restoration of blood flow to the kidney for 30 min of reperfusion. Additionally, some animals were subjected to a sham-operation as a control. The kidneys were subsequently extirpated from each rat at the end of experiment. The tissues were quickly frozen and kept at −80 °C until analysis. Eight rats were used for each experimental group.

Kidneys were homogenized in 0.2 M sodium phosphate pH 6.25 buffer (1:20, w/v) in a Potter–Elvehjem homogenizer fitted with a Teflon pestle. Homogenates were centrifuged at 10,000×g for 1 h and the supernatants were obtained. The supernatants were stored at −20 °C and utilized for biochemical analyses. Blood was collected from abdominal aorta into dried tubes and centrifuged at 4 °C, 1,000 g for 15 min.

### 4.4. Renal Pathological Examinations

At the end of each experiment, a portion of the kidney was removed, fixed in 4% formaldehyde for 24 h, and then embedded in paraffin. Sections were cut by a microtome at 4 μm thickness, mounted on a glass slide and stained with haematoxylin/eosin (H&E). The histological sections were examined with a light microscope to evaluate the kidney pathological changes.

### 4.5. Biochemical Analysis

Serum creatinine (SCr) and blood urea nitrogen (BUN) concentrations were measured using diacetyl monoxime and basic picric acid as substrates, respectively. Interleukin 6 (IL 6), Interleukin 8 (IL 8) and Tumor Necrosis Factor alpha (TNF-α) were analysed using commercially available kits (ELISA kits).

Lipid peroxidation MDA, as an end product of fatty acid peroxidation, was detected in kidney homogenates by thiobarbituric acid reactivity assay as previously described [41]. The total protein concentration was measured by the method of Lowry *et al.* [42].

The activity of SOD was assayed as described by Kakkar *et al.* [43]. A unit of the enzyme activity was defined as the enzyme reaction giving 50% inhibition of NBT reduction in 1 min under the assay conditions and expressed as units/mg protein.

Catalase activity was determined according to Lartillot *et al.* [44] with little modification as described by Bergmeyer [45]. CAT activity was determined spectrophotometrically at 240 nm using a specific absorption coefficient at 0.0392 cm^2^·μmol^−1^ H_2_O_2_. Briefly, substrate solution (2.5 mL) was made up by using 25 mM H_2_O_2_ in a 75 mM phosphate buffer (pH 7.0). supernatant (20 μL, containing >0.2 mg protein/mL) were mixed at 25 °C for 2 min and reaction was stopped by adding 1 M HCl (0.5 mL). CAT activity was calculated as μmol H_2_O_2_ decomposed/mg protein/min.

Glutathione peroxidase activity was measured by the use of consecutive glutathione reductase reaction and oxidation of NADPH, with t-butyl hydroperoxides as substrate [46]. A unit of GSH-Px activity was defined as the amount of GSH-Px needed to reduce initial glutathione concentration which was calculated according to Flohè and Gunzler [47] and expressed as unit per mg protein.

### 4.6. Statistical Analysis

All results were reported as mean ± S.E.M. The results were further analyzed using Student’s t-test to calculate significance of results.

## 5. Conclusions

From this investigation, we could verify that *Salvia miltiorrhiza* ethanol extract protects the kidney against ischemia-reperfusion damage by reducing Scr, BUN, IL-6, IL-8, TNF-α and MDA levels, increasing GSH and antioxidative enzyme activities. Furthermore, the protective activity was higher with increasing oral dose. The findings of the current study illustrate that *Salvia miltiorrhiza* ethanol extract, with its potent free radical scavenging and antioxidant properties, seems to be a highly promising agent in protecting renal tissue against oxidative damage and in preventing renal dysfunction due to ischemia/reperfusion.
